# Increased Serum S100B Levels in Patients With Epilepsy: A Systematic Review and Meta-Analysis Study

**DOI:** 10.3389/fnins.2019.00456

**Published:** 2019-05-16

**Authors:** Kai-Ge Liang, Rong-Zheng Mu, Yu Liu, Dan Jiang, Tian-Tian Jia, Yao-Jiang Huang

**Affiliations:** ^1^College of Life and Environmental Sciences, Minzu University of China, Beijing, China; ^2^Beijing Engineering Research Center of Food Environment and Public Health, Minzu University of China, Beijing, China; ^3^College of Equipment Management and UAV Engineering, Air Force Engineering University, Xian, China; ^4^Jarud Banner Agriculture and Animal Husbandry Bureau, Tongliao, China; ^5^Department of Environmental Health, Harvard T. H. Chan School of Public Health, Boston, MA, United States

**Keywords:** serum, S100B, level, epilepsy, meta-analysis

## Abstract

**Importance:** Accumulating evidence suggests that serum levels of S100B may play a role in epilepsy.

**Objective:** We performed a meta-analysis to quantitatively summarize the serum S100B data available for patients with epilepsy.

**Data source:** Two independent researchers conducted a systematic investigation of the Harvard Hollis+, Open Gray, Clinicaltrials, Wanfangdata, and CNKI databases through Dec 6, 2018, for all studies published in English and Chinese. The search terms included S100B and calcium-binding protein B in combination with epilepsy.

**Study selection:** Original studies and reported data from these search terms are included. Studies where data overlapped with other studies were excluded.

**Data extraction and synthesis:** investigators extracted, pooled and analyzed data from the included studies using a fixed-effects model in the Comprehensive Meta-Analysis3.3 and R software.

**Main outcomes and measures:** Peripheral blood levels of S100B in patients with epilepsy compared with controls. Aberrations in peripheral blood levels of S100B were hypothesized to be related to epilepsy.

**Results:** a fixed-effects meta-analysis of all 18 studies, including 1,057 unique participants, indicated that patients with epilepsy had significantly increased peripheral blood levels of S100B compared to controls (Hedges *g* = 1.568, 95% CI =1.431–1.706, *P* < 0.001). Sensitivity analysis showed that no single study significantly influenced the overall association of peripheral blood levels of S100B and epilepsy. Most of the subgroup analyses, including those of country, assay type and publication language, demonstrated a statistically significant association between peripheral blood levels of S100B and epilepsy. Meta-regression analyses indicated that gender (regression coefficient [SE], −0.2524 [0.0641]; 95%CI, −0.3781 to −0.1267; *P* = 0.0001) and mean age (regression coefficient [SE], −0.1224 [0.0426]; 95% CI, −0.2058 to −0.0390; *P* = 0.0040) might present serum S100B reductions, but sample size, years, assay type, publication language and country did not show moderating effects on the effect sizes. Furthermore, the trim-and-fill method used to adjust for funnel plot asymmetry in our meta-analysis confirmed that a positive outcome is unlikely to be due to publication bias.

**Conclusion and relevance:** the results of this meta-analysis provide evidence for a significant increase in serum S100B levels in patients with epilepsy. Serum S100B is the most worthwhile biomarker of epilepsy, which is helpful for the clinical diagnosis and prognosis of epilepsy.

## Introduction

Epilepsy is a chronic disorder of the brain that affects people of all ages and is defined as having two or more unprovoked seizures. Approximately 50 million people worldwide have epilepsy, making it one of the most common neurological diseases globally. An estimated 2.4 million people are newly diagnosed with epilepsy each year (http://www.who.int/en/news-room/fact-sheets/detail/epilepsy) (WHO, 2018). Identification of biomarkers that can help in guiding diagnosis and therapy has therefore been at the center stage of research efforts in the last years (Pitkänen et al., 2016). This is particularly true for anti-epileptogenic treatments, which would ideally require predicting which individual patients with an initial injury will later develop epilepsy. The consensus definition of the International League Against Epilepsy defines a biomarker for epileptogenesis as an objectively measurable characteristic of a biological process that reliably identifies the development, presence, severity, progression, or localization of an epileptogenic abnormality (Pitkänen et al., 2016).

The misdiagnosis rate of seizures is relatively high, and currently there is a lack of biomarkers to reliably diagnose epilepsy (Walker et al., 2016). Therefore, finding specific markers related to epilepsy is important for assessing the severity of seizure and whether there is epilepsy (Hao et al., 2017). Ideally, a set of biomarkers is used to assess the duration of the epileptic seizure and the interictal period following injury. Due to their advantages, minimally invasive and peripheral biomarkers play an important role in the evaluation of brain diseases such as epilepsy. Biomarkers associated with brain inflammation, growth factors, microRNAs, oxidative stress, and metabolic dysfunction may contribute to the early diagnosis of epilepsy (Pitkänen et al., 2016). A clinically meaningful ideal peripheral biomarker should meet the following conditions: (1) This protein or molecule is low or even undetectable in normal serum; (2) it exists in the brain and CSF, and has a higher concentration in the brain parenchyma than in plasma; (3) it can exudate when the blood–brain barrier (BBB) is opened; and (4) it can be further detected in brain injury, such as with increased reactive glia. It can be further released by brain cells when the brain is damaged, such as increased reactive neuroglia.

S100B is a Ca2+-binding protein and is expressed primarily by astrocytes. S100 proteins are involved in the regulation of a number of cellular processes such as signal regulating, cell cycle progression, and act as a stimulator of cell proliferation and migration and as an inhibitor of apoptosis and differentiation (Donato et al., 2009; Riuzzi et al., 2018; Michetti et al., 2019). S100B meets all the above characteristics of a clinically meaningful ideal peripheral biomarker (Walker et al., 2016). Serum S100B is the most worthwhile biomarker of brain damage (Marchi et al., 2004) and disorders of the Nervous System. It was found that the level of serum S100B in epileptic patients or infants with epileptic seizure was significantly increased, which is helpful for the clinical diagnosis of epileptic seizures (Steinhoff et al., 1999; Sendrowski et al., 2004; Kacinski et al., 2012). Functional assessment of BBB status was widely accepted as the gold standard for BBB permeability by calculating CSF-serum albumin quotient (QA) and contrast-enhanced MRI (Hoffmann et al., 2011). Some studies have reported that serum S100B is associated with QA, allowing CSF protein to be measured indirectly through S100B without the spinal puncture. Studies have shown that the negative predictive value of S100B is equivalent to enhanced MRI (Blyth et al., 2009).

Although numerous studies have reported that serum S100B levels are associated with epileptic seizures (Steinhoff et al., 1999; Portela et al., 2003; Sendrowski et al., 2004; Ravizza et al., 2017; van Vliet et al., 2017), epidemiological studies on this topic remain controversial due to inconsistent clinical studies. Therefore, to our knowledge, we conducted the first systematic review and meta-analysis, quantitatively summarized the peripheral blood S100B level in epileptic patients and healthy controls (HC) to study the correlation between epilepsy and serum S100B levels, and performed meta-regression and subgroup analysis to adjust for confounding factors that may affect the outcome.

## Materials and Methods

### Search Strategy and Study Selection

Two researchers independently conducted a systematic search peer-reviewed English and Chinese literature, including comprehensive searches of Harvard's HOLLIS+ (Harvard's Hollis+ includes databases such as PubMed, PsycINFO, and Web of Science), Open Gray, Clinicaltrials, the Wanfangdata and China National Knowledge Infrastructure databases (CNKI) through Dec 6, 2018, for all studies published in English and Chinese. The English database search consisted of the following terms: (S100β OR S100B OR S100B Protein OR Calcium-binding protein B) AND (Epilepsy OR Seizures). The Chinese database search consisted of the following terms: (钙结合蛋白β OR 钙结合蛋白B OR S100B) and (癫痫). Original studies reporting data on serum or plasma S100B levels in patients and HC with epilepsy were included. The exclusion criteria of this meta-analysis were as follows: overlapping data or no necessary data. This study followed the guidelines from the Cochrane Handbook for Systematic Reviews of Interventions Version 5.1.0 (Higgins and Green, 2011).

### Data Extraction

Data on sample sizes, mean S100B concentrations, and s.d. were extracted from the included studies as primary outcomes to generate effect sizes by two independent investigators. Information was also extracted on publication year, region (country), mean age, gender distribution (male%), diagnosis, medication, disease duration, the Positive and Negative Symptoms Scale (PANSS) total score, assay type, and sample source for potential moderator analyses (Table S1). We contacted the corresponding authors to request the necessary data when the records did not provide sufficient information to generate effect sizes.

### Statistical Analysis

We performed all statistical analyses used the Comprehensive Meta-Analysis software (Version 3.3. Biostat, Englewood, NJ, USA, 2014) (R Studio, 2014), and RStudio (Version1.1.442– © 2009–2018 RStudio, Inc.) (R Studio, 2015). Effect sizes were calculated as standardized mean difference generated by sample sizes, mean S100B concentrations and s.d. in S100B levels between epilepsy patients and HC subjects, and standardized mean difference converted to Hedges g, which provides a correction factor that addresses the potential biases derived from a small sample size. We used a fixed-effects models to evaluate the effect size and a 95% confidence interval (95% CI) to analyze the statistical difference for all studies combined. The actual effect size was affected by confounding factors within and between studies; therefore, we chose a fixed-effects model to perform a meta-analysis.

We used a sensitivity analysis via a leave-one-out study (R Studio, 2014) to evaluate whether an individual study influenced the results of the meta-analysis. Heterogeneity among studies was evaluated by the Cochran Q test and the *I*^2^ statistic. The impact of heterogeneity among studies was calculated by the *I*^2^ values, with *I*^2^ values of 25, 50, and 75% indicating low, moderate, and high heterogeneity, respectively. In addition, we evaluated the relevant covariates by a meta-regression analysis of potential moderating effects, including publication year, sample size, country, mean age, disease severity (PANSS score), assay type, publication language, and gender. We conducted subgroup analyses by region, assay type, and publication language.

The funnel plot used to assess publication bias was performed with the Comprehensive Meta-Analysis software (Version 3.3. Biostat, Englewood, NJ, USA, 2014), and the statistical significance was determined by Egger's test. We examined publication bias using the trim-and-fill method. Further, the potential effect of publication bias was assessed by a Classic fail-safe N test, which calculates the number of missing studies that would need to be added to the analysis to produce statistically insignificant results. All statistical analyses were conducted with the Comprehensive Meta-Analysis software (Version 3.3. Biostat, Englewood, NJ, USA, 2014) (R Studio, 2014) and RStudio (Version1.1.442– © 2009-2018 RStudio, Inc.) (R Studio, 2015).

## Results

**Figure 1:**
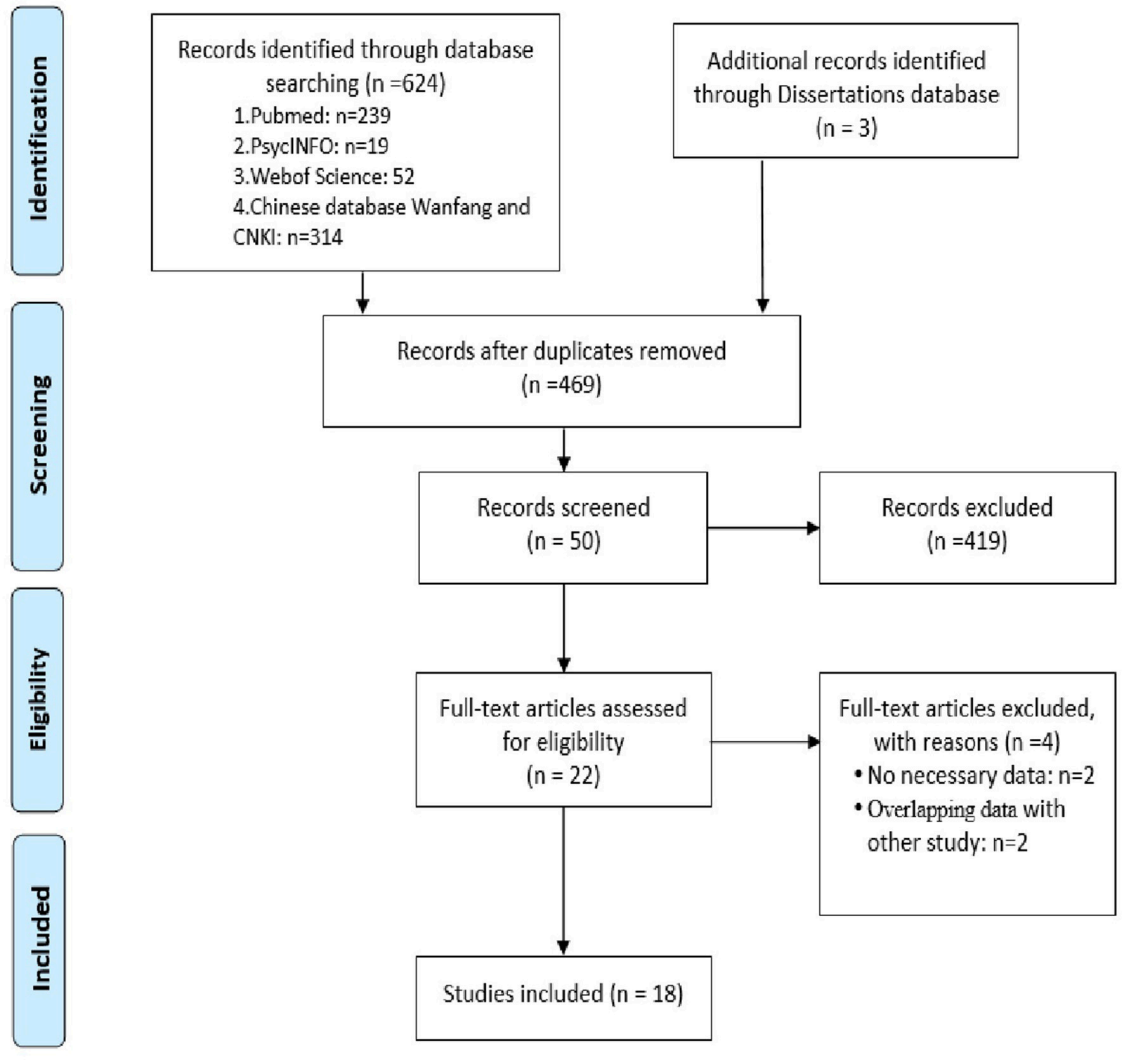
PRISMA flowchart. PRISMA flowchart for the literature search.

We carried out a systematic search that produced 310 records from the English databases in HOLLIS+ (Harvard's Hollis + includes databases such as PubMed, PsycINFO, and Web of Science), Open Gray and Clinicaltrials, 314 records from Chinese databases by Wanfang and CNKI, and three dissertations from HOLLIS+ and CNKI. We examined abstracts of the selected articles and retrieved and reviewed the full text of those that were possibly eligible. Twenty-two records were identified for full-text analysis. We scrutinized these records and excluded four due to a lack of necessary data (Yelmo et al., 2012; Chen et al., 2015), because the results were also reported in other publications (Lu, 2010; Chen, 2011a). Ultimately, 18 studies encompassing 1,057 cases and 821 controls met the criteria for inclusion in our meta-analysis (Figure 1) (Portela et al., 2003; Li et al., 2004; Yun, 2009; Lu et al., 2010; Chen, 2011b; Liu et al., 2011; Atici et al., 2012; Mikkonen et al., 2012; Shiihara et al., 2012; Wang and Han, 2012; Xu et al., 2012; Yuan et al., 2014; Yun et al., 2015; Wang et al., 2016, 2018; Hao et al., 2017; Zhao et al., 2017; Zhang et al., 2018).

### Main Association of S100B Levels With Epilepsy

**Figure 2:**
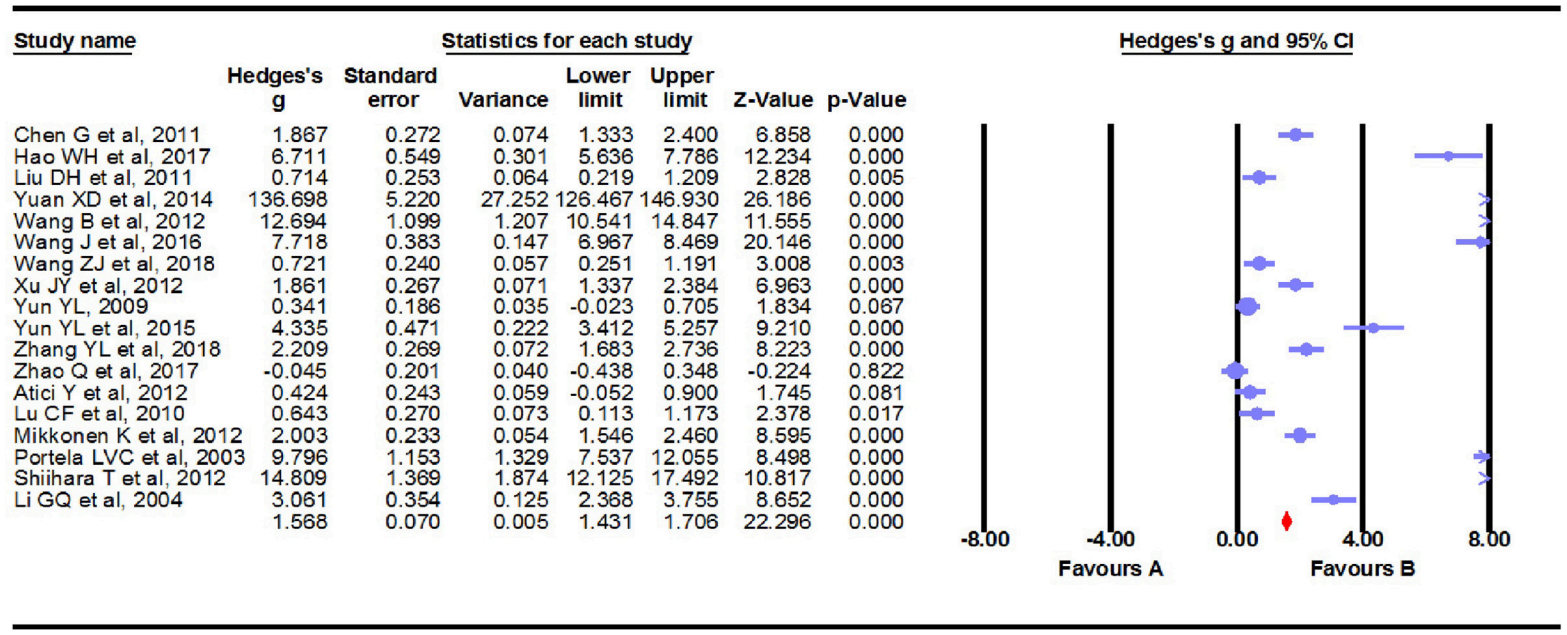
Forest plot for association between serum S100B levels and epilepsy. Square sizes are proportional to study weights. The diamond marker indicates pooled effect size.

**Figure 3:**
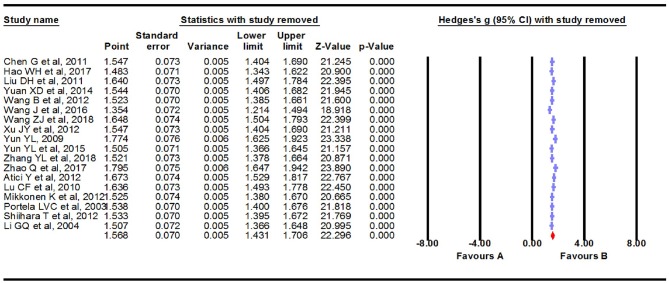
Sensitivity analysis. No single study significantly influenced the overall association of S100B levels with epilepsy by sensitivity analysis.

We carried out a meta-analysis using a fixed-effects model on the 18 extracted studies. A forest plot is shown in Figure 2, illustrating that serum S100B levels were significantly increased in patients with epilepsy (Hedges *g* = 1.568, 95% CI = 1.431–1.706, *P* < 0.001). Sensitivity analysis is conducted by excluding one study at a time to assess whether a particular study is responsible for the results of the meta-analysis. The results showed that no single study significantly influenced the overall association of S100B levels with epilepsy by sensitivity analysis (Figure 3). Nevertheless, we found significant heterogeneity among the studies in our meta-analysis (Q = 492.695, degree of freedom (df) = 17, *I*^2^ = 98.861, *P* < 0.001). Moreover, none of the single studies fully explained heterogeneity, which was high in all studies.

### Subgroup Analyses

**Figure 4:**
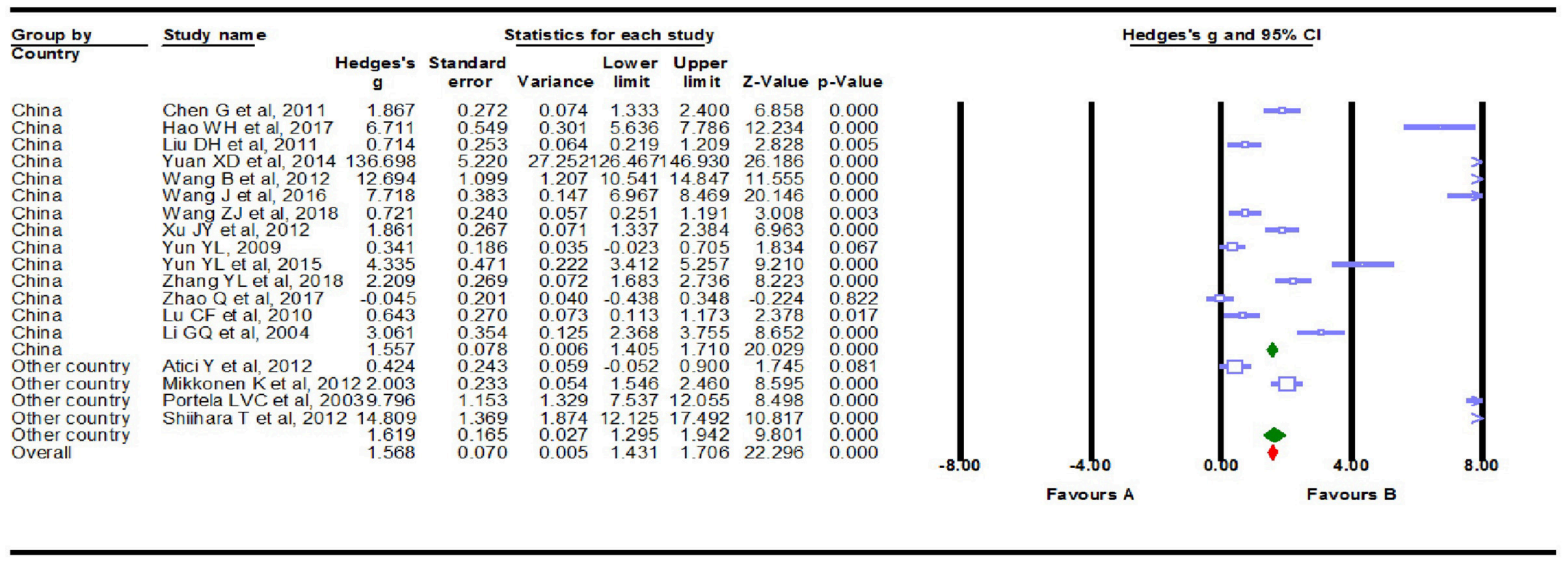
Forest plot for subgroup analysis. Subgroup analysis by country.

**Figure 5:**
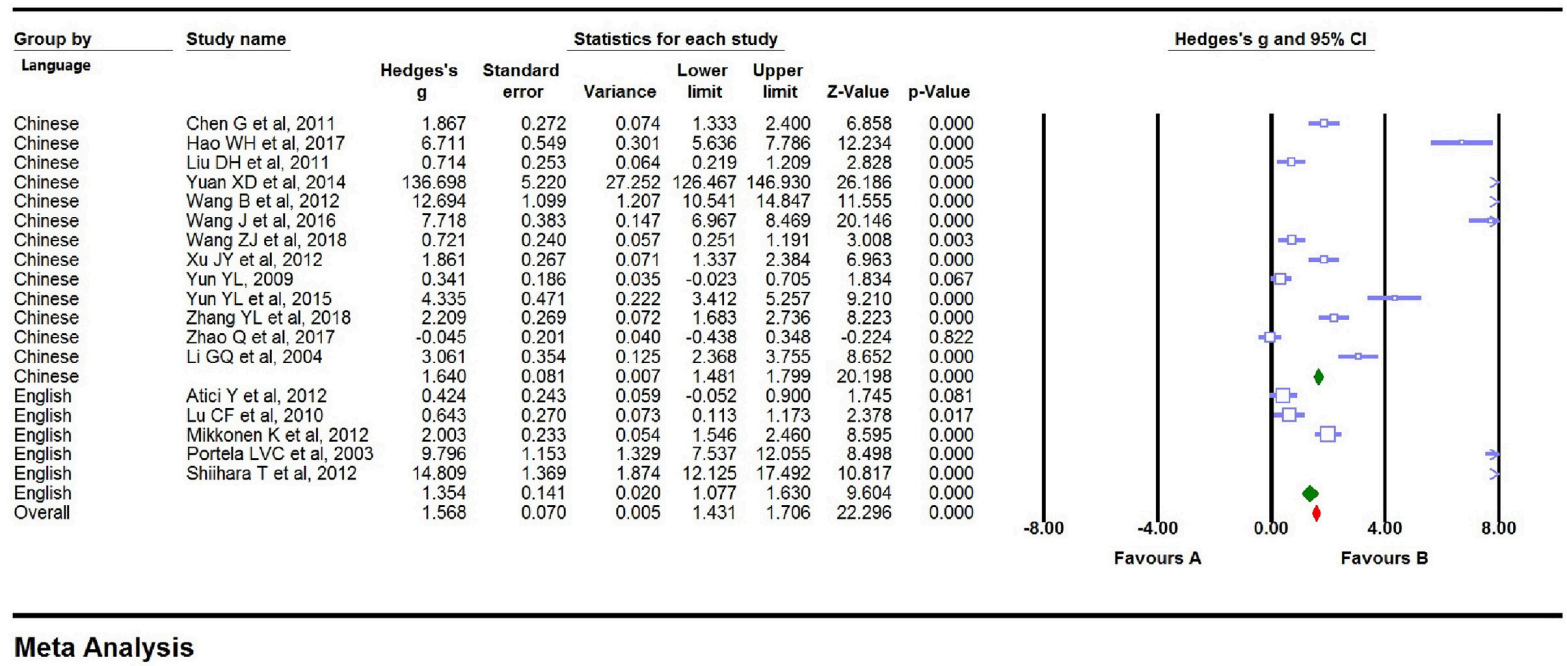
Forest plot for subgroup analysis. Subgroup analysis by language.

**Figure 6:**
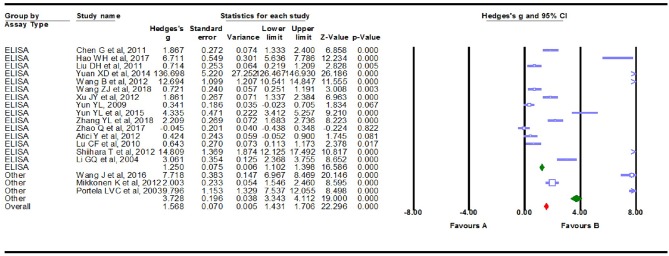
Forest plot for subgroup analysis. Subgroup analysis by assay type.

We conducted subgroup analyses to explore the potential clinical moderators and the possible sources that explained the large heterogeneity. Fourteen of the Eighteen studies (Li et al., 2004; Yun, 2009; Lu et al., 2010; Chen, 2011b; Liu et al., 2011; Wang and Han, 2012; Xu et al., 2012; Yuan et al., 2014; Yun et al., 2015; Wang et al., 2016, 2018; Hao et al., 2017; Zhao et al., 2017; Zhang et al., 2018) in the meta-analysis were from China, and the remaining four studies (Portela et al., 2003; Atici et al., 2012; Mikkonen et al., 2012; Shiihara et al., 2012) were from other countries. Studies from China showed a marginally significant association (Hedges *g* = 1.557, 95% CI = 1. 405–1.710, *P* < 0.001), while the additional studies showed highly significant results (Hedges *g* = 1.619, 95% CI = 1.295–1.942, *P* < 0.001, Figure 4). High levels of heterogeneity among studies were still found in China's 14 studies [*Q* = 322.498, degree of freedom (df) = 13, *I*^2^ = 99.017, *P* < 0.001] and the other four studies [*Q* = 170.084, degree of freedom (df) = 3, *I*^2^ = 98.236, *P* < 0.001]. Then, the summary Hedges *g* (95% CI) for studies retrieved from the English (Portela et al., 2003; Lu et al., 2010; Atici et al., 2012; Mikkonen et al., 2012; Shiihara et al., 2012) and Chinese (Li et al., 2004; Yun, 2009; Chen, 2011b; Liu et al., 2011; Wang and Han, 2012; Xu et al., 2012; Yuan et al., 2014; Yun et al., 2015; Wang et al., 2016, 2018; Hao et al., 2017; Zhao et al., 2017; Zhang et al., 2018) literature was 1.354 (1.077–1.630) and 1.640 (1.481–1.799), respectively. We found high levels of heterogeneity among articles retrieved from the English [*Q* = 179.564, degree of freedom (df) = 4, *I*^2^ = 97.772, *P* < 0.001] and Chinese literature [Q = 310.004, degree of freedom (df) = 12, *I*^2^ = 99.084, *P* < 0.001] (Figure 5). Further analysis of assay studies by ELISA (Li et al., 2004; Yun, 2009; Lu et al., 2010; Chen, 2011b; Liu et al., 2011; Atici et al., 2012; Shiihara et al., 2012; Wang and Han, 2012; Xu et al., 2012; Yuan et al., 2014; Yun et al., 2015; Hao et al., 2017; Zhao et al., 2017; Wang et al., 2018; Zhang et al., 2018) or other (Portela et al., 2003; Mikkonen et al., 2012; Wang et al., 2016) showed that compared to healthy controls, serum S100B levels significantly increased in patients with Epilepsy (Figure 6, Hedges *g* = 1.250, 95% CI = 1.102–1.398, *P* < 0.001 and Hedges *g* = 3.728, 95% CI = 3.343–4.112, *P* < 0.001). Again, we observed high levels of heterogeneity among assay studies by ELISA [Q = 162.742, degree of freedom (df) = 14, *I*^2^ = 98.796, *P* < 0.001] or other [Q = 190.959, degree of freedom (df) = 2, *I*^2^ = 98.953, *P* < 0.001].

### Meta-Regression Analyses (Investigation of Heterogeneity)

**Figure 7:**
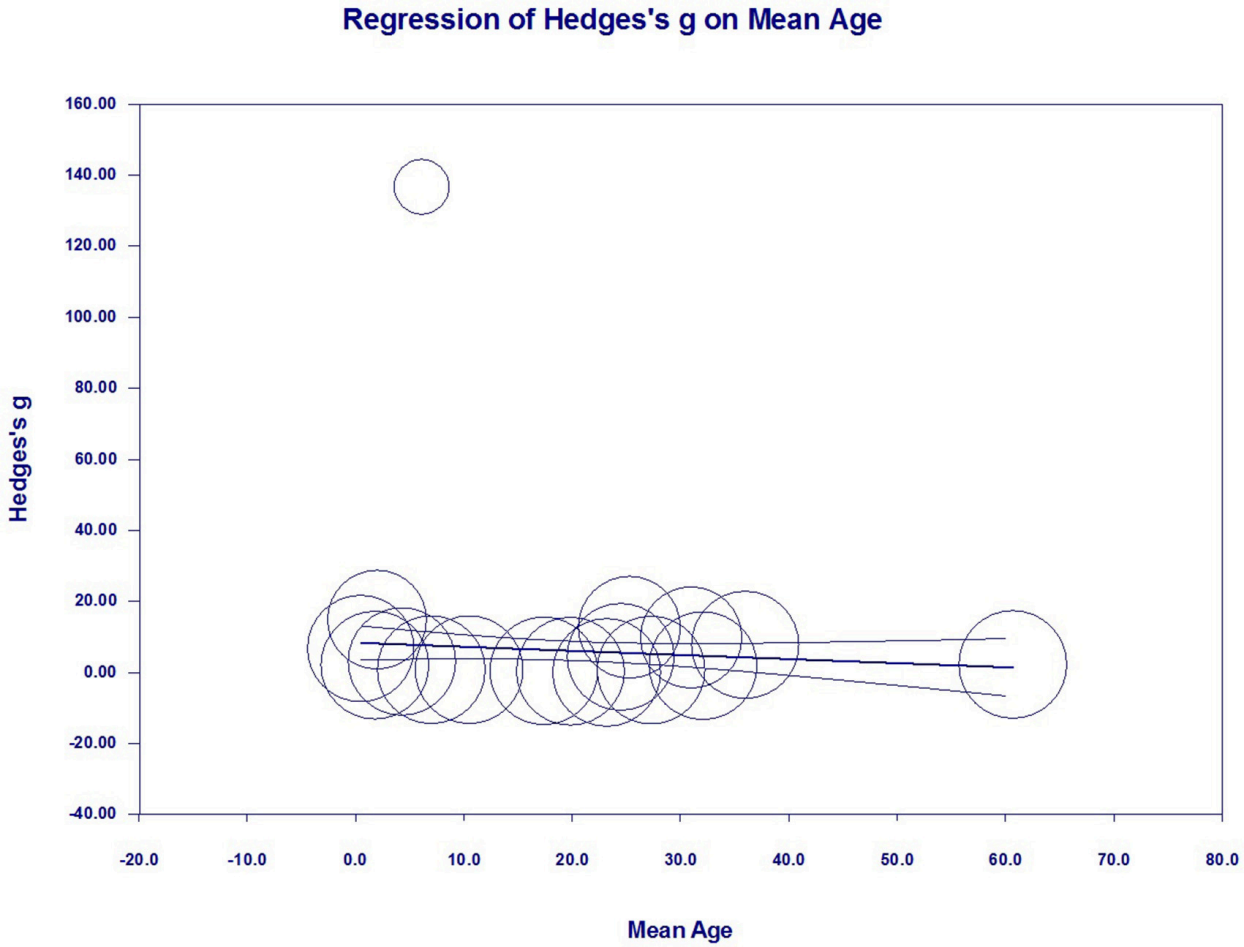
Meta-regression in all studies. Association between mean age and effect size (Hedges *g*).

**Figure 8:**
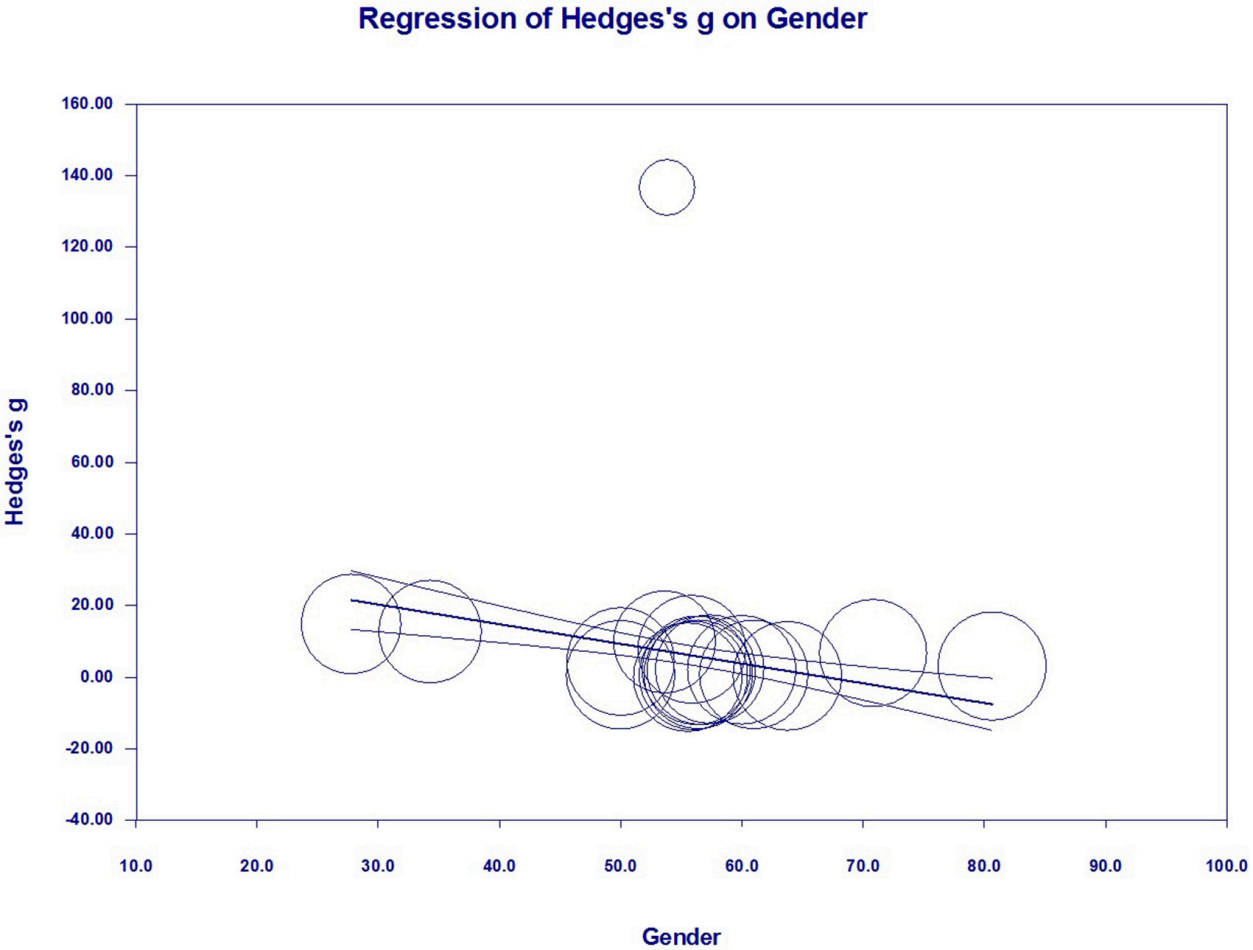
Meta-regression in all studies. Association between gender and effect size (Hedges *g*).

To investigate the possible variables explaining the high levels of heterogeneity among studies, a meta-regression analysis was performed that included publication year, sample size, mean age, assay type, publication language, gender, and country of each study. Of these possible variables, mean age (regression coefficient [SE], −0.1224 [0.0426]; 95% CI, −0.2058 to −0.0390; *P* = 0.0040) (Figure 7) and gender (regression coefficient [SE], −0.2524 [0.0641]; 95% CI, −0.3781 to −0.1267; *P* = 0.0001) (Figure 8) correlated with effect sizes, suggesting that higher mean age and gender might present serum S100B reductions. Sample size, publication year, assay type, publication language, and country did not show moderating effects on the effect sizes by meta-regression analysis.

### Publication Bias

**Figure 9:**
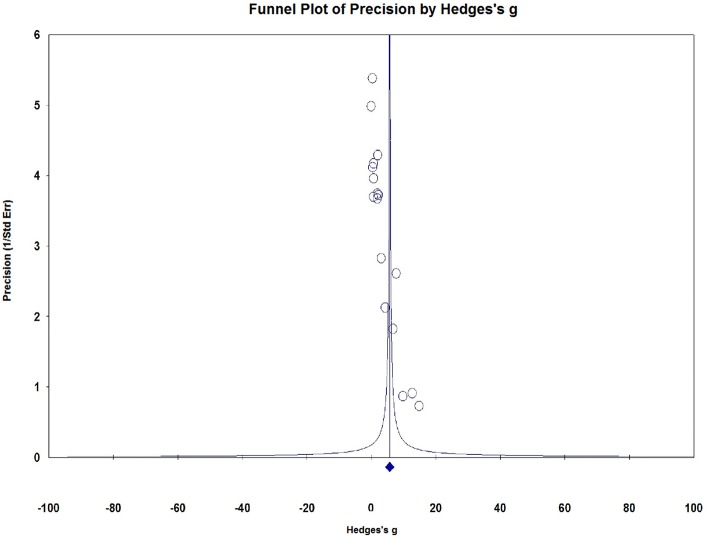
Funnel plots for assessment of publication bias. Funnel plots for assessment of publication bias.

**Figure 10:**
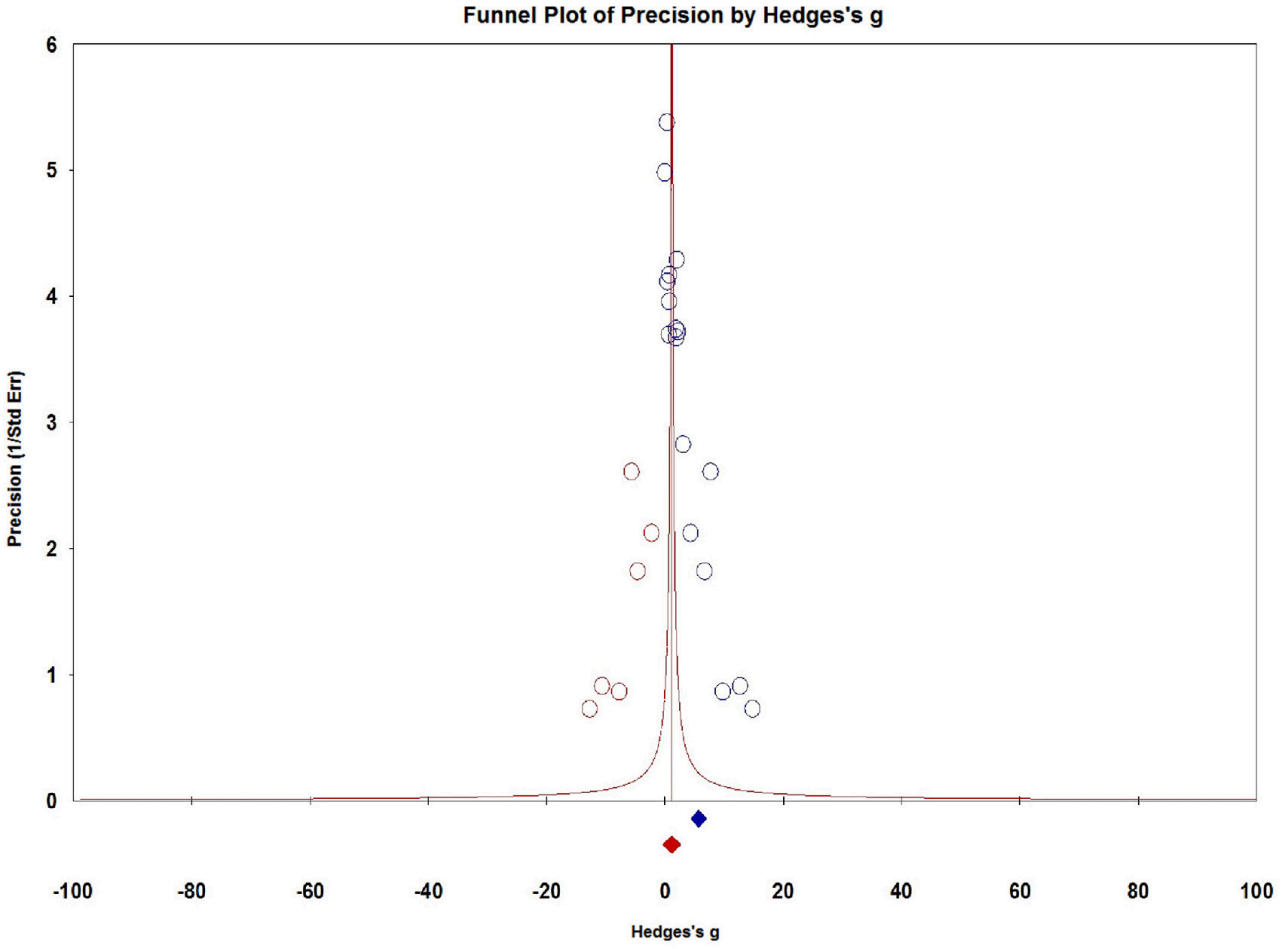
Funnel plots for assessment of publication bias. The trim-and-fill method to adjust for funnel plot asymmetry in our meta-analysis.

Visual inspection of dots appeared to be non-symmetric, which suggested publication bias (Figure 9). We further investigated publication bias using the trim-and-fill method to adjust for funnel plot asymmetry in our meta-analysis, which yielded significant results (Hedges *g* = 1.034, 95% CI = 0.902–1.167, *P* < 0.001, Figure 10), thus confirming that the significant association between Serum S100B levels and epilepsy in this meta-analysis is unlikely to be caused by publication bias.

## Discussion

To our knowledge, this is the first meta-analysis performed on peripheral blood S100B levels in patients with epilepsy. We integrated and analyzed a number of individual studies, and we found that patients with epilepsy had significantly increased peripheral blood levels of S100B compared to controls. Although epidemiological studies on this topic remain controversial due to inconsistent clinical studies, our meta-analysis provides evidence of significantly increased peripheral blood levels of S100B in patients with epilepsy. Sensitivity analysis indicated that our results were not significantly influenced by any individual study. Subgroup analyses demonstrated a statistically significant association between peripheral blood levels of S100B and epilepsy. We adjusted for publication bias by using the trim-and-fill method, and no significant effect on publication bias was found, suggesting that our results are unlikely to be due to publication bias.

Although there is considerable heterogeneity between studies in this meta-analysis, the strength of this work is the use of meta-regression and subgroup analyses to adjust possible moderators. Subgroup analyses that included assay type, country, and publication language showed that none of our subgroups fundamentally reduced heterogeneity, although all subgroup analyses showed a significant increase in serum S100B levels in patients with Epilepsy. We conducted a meta-regression analysis including publication year, sample size, mean age, assay type, publication language, gender, and country. The results suggest that mean age and gender may be related to the effect size, which is reasonable as the level of S100B may be associated with the effect size as the mean age and proportion of males increase. Furthermore, the trim-and-fill method used to adjust for funnel plot asymmetry in our meta-analysis confirmed that a positive outcome is unlikely to be due to publication bias.

The consensus definition of the International League Against Epilepsy defines a biomarker for epileptogenesis as an objectively measurable characteristic of a biological process that reliably identifies the development, presence, severity, progression, or localization of an epileptogenic abnormality (Pitkänen et al., 2016). Serum S100B is the most worthwhile biomarker of brain damage and disorders of the nervous system. S100B meets all the clinically meaningful ideal peripheral biomarker characteristics (Walker et al., 2016). It was found that the level of serum S100B in epileptic patients or animal models of epilepsy was significantly increased (Griffin et al., 1995; Steinhoff et al., 1999; Sendrowski et al., 2004; Hou et al., 2012; Wei, 2014; Ravizza et al., 2017), and an animal model showed that oral antiepileptic reagent reduces kainic acid-induced epileptic seizures and neuronal death accompanied by attenuating glial cell proliferation and S100B proteins (Fang et al., 2006; Lin and Hsieh, 2011; Tang et al., 2017), which is helpful for the clinical diagnosis and prognosis of epilepsy (Wang, 2011; Freund et al., 2015; Heidari et al., 2015; Lee et al., 2017). Serum S100B is associated with QA, allowing CSF protein to be measured indirectly through S100B without the spinal puncture. Studies have shown that the negative predictive value of S100B is equivalent to enhanced MRI (Blyth et al., 2009). Due to the advantages of being minimally invasive, S100B plays an important role in the evaluation of brain diseases such as epilepsy and may contribute to early diagnosis, crucial medical intervention before irreversible damage, and the evaluation of therapeutic effects and prognosis of epilepsy (Zongo et al., 2012).

Despite its significant strengths, this meta-analysis has several limitations. First, we used a fixed-effects model to alleviate the huge heterogeneity between studies in the meta-analysis, but the limitation of this model is that it does not strictly rule out the effects of heterogeneity (Alexander et al., 2002). In fact, the statistical significance of the results of the fixed effect model and the random effect model does not change much, but the value of the effect size of the random effect model is larger (Hedges *g* = 1.568, 95% CI = 1.431–1.706 in fixed-effect model; Hedges *g* = 5.687, 95% CI = 4.327–7.048 in random-effect model) in this meta-analysis. Second, since most of the data come from case-control studies, the data may be subject to selection bias, measurement bias, and interviewer bias. Last, we did not stratify the different types of epilepsy and pharmacological history of the patients because of the paucity of studies and data. However, studies have shown that the level of S100 varies according to the type of epilepsy (Liu et al., 2011; Hao et al., 2017) and pharmacological history of the patients (Li, 2012; Tang et al., 2017). We hope to make additional stratification analysis in future.

## Conclusion

In conclusion, our meta-analysis provides evidence that Serum S100B levels were significantly elevated in patients with epilepsy. Serum S100B is the most worthwhile biomarker of epilepsy, which is helpful for the clinical diagnosis and prognosis of epilepsy.

## Data Availability

All datasets generated for this study are included in the manuscript and/or the Supplementary Files.

## Author Contributions

Y-JH: study conception and design. Y-GL, YL, and R-ZM: data collection. Y-JH, DJ, and T-TJ: analysis or interpretation of data. All authors drafted the manuscript and critical revision.

### Conflict of Interest Statement

The authors declare that the research was conducted in the absence of any commercial or financial relationships that could be construed as a potential conflict of interest.

## Supplementary Material

Table S1 Extracted about the publication year, region (country), mean age, gender distribution (male %), diagnosis, medication, disease duration, the Positive and Negative Symptoms Scale (PANSS) total score, assay type, and sample source for potential moderator analyses.
