# Supplementary material for: Increased Serum S100B Levels in Patients With Epilepsy: A Systematic Review and Meta-Analysis Study
Source: Front Neurosci. 2019 May 16;13:456. doi: 10.3389/fnins.2019.00456 (PMC6532535; doi:10.3389/fnins.2019.00456)
Supplement: Table S1 — Extracted about the publication year, region (country), mean age, gender distribution (male %), diagnosis, medication, disease duration, the Positive and Negative Symptoms Scale (PANSS) total score, assay type, and sample source for potential moderator analyses. [file Data_Sheet_1.PDF]

Study	Year	Region/Country	Samples Size(Case/HC)	Mean Age(Case/HC)	Gender (%	Mean disease duration (year)	Mean PANSS score	Sample Source	Assay type	Serum, S100B Concentrations ($\bar{X}$ ±S) （Case/HC, ng /ml）
Chen G et al. 2011	2011	Jiangshu,China	30/50	32.1± 14.6/33.9± 11.4	56.666	NA	NA	Serum	EL ISA	1.6756± 0.4903/0.8892± 0.3678
Hao WH et al. 2017	2017	Hebei, China	48/40	1-11moonth/1.5-12	70.833	NA	NA	Serum	ELISA	1.23369 ± 0.20847/0.16844± 0.04516
Liu DH et al. 2011	2011	Guangdong, China	82/20	7. 01 ± 4. 05/7. 6 ± 3. 28	60.98	NA	NA	Serum	ELISA	0.87 ± 1. 06/0.16 ± 0.58
Yuan XD et al. 2014	2014	Zhejiang, China	208/135	6.13 ± 5.02/ 5.09 ± 4.16	53.846	NA	NA	Serum	ELISA	0.29008± 0.001/0.15380 ± 0.001
Wang B et al. 2012	2012	Liaoning, China	35/35	25.3± 4.7/25.2±4.9	34.286	12.1±2.5		Serum	ELISA	1.52±0.11/0.23± 0.09
Wang J et al. 2016	2016	Shandong, China	34/34	35.99 ± 16.64/36.17 ± 16.93	55.882	3months-21Years	NA	Serum /CSF	RT-PCR Western Blot	0.911 ± 0.112/0.032 ± 0.115
Wang ZJ et al. 2018	2018	Beijing,China	39/34	７～１４/８～１４	56.41		NA	Serum /CSF	EL ISA	1.86 ±0.24/0.52 ± 0.08
Xu JY et al. 2012	2012	Jiangshu,China	32/50	32.1 ± 14. 6/33. 4 ± 11.4	56.25	8.7 ± 7.8/7.7 ± 5.9	NA	Serum	ELISA	1.68 ± 0.49/0.89 ± 0.37

Yun YL. 2009	2009	Inner Mongolia, China	69/50	19.85/9.33	63.768	lday-20year	NA	Serum	ELISA	0.103±0.052/0.087±0.038
Yun YL et al. 2015	2015	Inner Mongolia, China	30/30	24 5± 4.5 /25± 4.8	50	NA	NA	Serum	ELISA	0.287±0.054/0.109±0.020
Zhang YL et al. 2018	2018	Helongjiang, China	68/30	69.7/55.3	57.353	NA	NA	Serum	ELISA	0. 31±0. 10/0. 11±0. 06
Zhao Q et al. 2017	2017	Inner Mongolia, China	63/40	23.2/NA	55.55	3.6/NA	NA	Serum	ELISA	0.1397±0.007827/0.1401±0.034
Atici Yeliz et al. 2012	2012	Ankara, Turkey	39//30	17.4-7.3/17.1-9.8	56	NA	NA	Serum	ELISA	0.0326±0.0078/0.0295±0.0078
Lu CF et al. 2010	2010	Beijing, China	28/28	27.3±7.7/26.4±8.4	50	NA/NA	NA	Serum	ELISA	0.42±0.20/0.31±0.13
Mikkonen Kirsi et al. 2012	2012	Oulu, Finland	103/33	1.8/1.4	60	NA	NA	Serum/CSF	Immunoluminometric assay	0.12±0.001 /0.11±0.01
Portela L.V.C et al. 2003	2003	RS(Rio Grande do Sul), Brazil	19/20	32.7 ± 9.1/33.9 ± 12.0	53.631	11.3 (8.9)/13.6 (9.8)/NA	NA	Serum	Immunoluminometric assay	0.005±0.001/0.016±0.001
Shiihara Takashi et al. 2012	2012	Gunma, Japan	18/43	1.0/1.0	27.777	5.0/0/1.0	NA	serum or CSF	ELISA	0.065.0±0.001/0.050±0.001

Guang-Qian L	2004	Zhejiang, China	31/38	4.3±3.1/4.1 ±2.8	80.645	NA	NA	Serum /CSF	ELISA	0.56±0.21/0.11± 0.05

Abbreviations: HC, Healthy Controls; ELISA, Enzyme-Linked ImmunoSorbent Assay; `X±S, `X, Mean of Concentrations; S=SD, standard deviation; CSF, Cerebrospinal Fluid; OR, Odds Ratio; RR, Risk ratio(Ie/Io) or Relative Risk ; 95%CI, 95% Confidence interval; RD, Risk Differnce; PANSS, Positive and Negative Symptoms Scale; NA, not available; S100B, Calcium-binding protein B
